# Stretch-Induced Biased Signaling in Angiotensin II Type 1 and Apelin Receptors for the Mediation of Cardiac Contractility and Hypertrophy

**DOI:** 10.3389/fphys.2020.00181

**Published:** 2020-03-13

**Authors:** Kinya Seo, Victoria N. Parikh, Euan A. Ashley

**Affiliations:** ^1^Division of Cardiovascular Medicine, Department of Medicine, Stanford University, Stanford, CA, United States; ^2^Department of Genetics, Stanford University, Stanford, CA, United States

**Keywords:** angiotensin II, AT_1_R, apelin, APJ, β-arrestin, Frank–Starling law, Anrep effect, cardiac hypertrophy

## Abstract

The myocardium has an intrinsic ability to sense and respond to mechanical load in order to adapt to physiological demands. Primary examples are the augmentation of myocardial contractility in response to increased ventricular filling caused by either increased venous return (Frank–Starling law) or aortic resistance to ejection (the Anrep effect). Sustained mechanical overload, however, can induce pathological hypertrophy and dysfunction, resulting in heart failure and arrhythmias. It has been proposed that angiotensin II type 1 receptor (AT_1_R) and apelin receptor (APJ) are primary upstream actors in this acute myocardial autoregulation as well as the chronic maladaptive signaling program. These receptors are thought to have mechanosensing capacity through activation of intracellular signaling via G proteins and/or the multifunctional transducer protein, β-arrestin. Importantly, ligand and mechanical stimuli can selectively activate different downstream signaling pathways to promote inotropic, cardioprotective or cardiotoxic signaling. Studies to understand how AT_1_R and APJ integrate ligand and mechanical stimuli to bias downstream signaling are an important and novel area for the discovery of new therapeutics for heart failure. In this review, we provide an up-to-date understanding of AT_1_R and APJ signaling pathways activated by ligand versus mechanical stimuli, and their effects on inotropy and adaptive/maladaptive hypertrophy. We also discuss the possibility of targeting these signaling pathways for the development of novel heart failure therapeutics.

## Introduction

**FIGURE 1:**
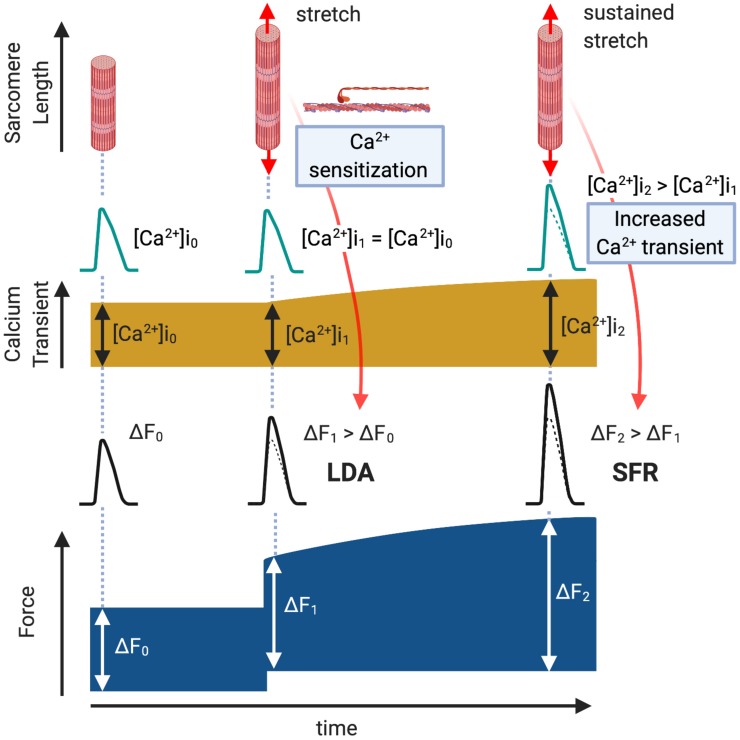
Length-Dependent Activation (LDA) and Slow Force Response (SFR). Immediately after a cardiac muscle is stretched, there is a rapid Ca^2+^-independent rise in developed force (ΔF_1_ > ΔF_0_, LDA) followed by a more gradual rise that is associated with an intracellular Ca^2+^ increase (ΔF_2_ > ΔF_1_, SFR).

The working heart adjusts cardiac output to changes in hemodynamic load in order to adapt to physiological demand. The first adaptation occurs immediately, on a beat-to-beat basis, after the ventricle is dilated by increased inflow. The relationship between end-diastolic volume and cardiac output, described by Ernest Henry Starling in a series of papers between 1912 and 1914, has been called “Starling’s Law of the Heart” or the “Frank–Starling relationship” which explains how the heart adapts to change in mechanical load by adjusting its contractile function (Katz Arnold, 2002). The main cellular mechanism that underlies the Frank–Starling relationship is enhanced myofilament sensitivity to Ca^2+^ at a longer sarcomere length, commonly referred to as length-dependent activation (Allen et al., 1974; de Tombe, 2003) (Figure 1, LDA). This length-dependent activation is modulated by posttranslational modification of myofilament proteins, such as cardiac troponin I (cTnI) (Tachampa et al., 2007; Wijnker et al., 2014), myosin-binding protein C (MyBPC) (Kumar et al., 2015), myosin regulatory light chain (Toepfer et al., 2016; Breithaupt et al., 2019) and titin (Hamdani et al., 2017). While the upstream molecular mechanisms that induce myofilament modifications and length-dependent activation had been poorly understood, recent studies suggest the roles of G protein-coupled receptors (GPCRs): angiotensin II type 1 receptor (AT_1_R) (Abraham et al., 2016) and apelin receptor APJ (Peyronnet et al., 2017; Parikh et al., 2018).

The myocardium has another autoregulatory mechanism to gradually increase its contractility in the setting of increased systemic resistance. Gleb Von Anrep first described in 1912 that the heart exhibits progressive increases in contractility in response to left ventricular dilation induced by aortic clamping *in vivo* (von Anrep, 1912). This “Anrep effect” had been interpreted as secondary to a neurohormonal effect or increased oxygen consumption due to the change in coronary perfusion [known as the “Gregg phenomenon” (Gregg and Shipley, 1944)] until Stanley Sarnoff and his colleagues reproduced this phenomenon in pressure/flow controlled isolated hearts and defined it as an autoregulation of myocardium (Sarnoff et al., 1960; Sarnoff and Mitchell, 1961). Subsequently, in isolated ventricular muscle strips exposed to sudden myocardial stretch, a gradual secondary increase in isometric/isotonic force was observed to follow the initial rise in contractility induced by the Frank–Starling mechanism (Parmley and Chuck, 1973). It has since been proposed that this “Slow Force Response (SFR)” (Figure 1, SFR) is the *in vitro* equivalent of the Anrep effect (Alvarez et al., 1999). Unlike the Frank–Starling mechanism, SFR is induced by a gradual increase in Ca^2+^ transient amplitude (Allen and Kurihara, 1982; Kentish and Wrzosek, 1998) through the activation of multiple intracellular components and ion transporters (Cingolani et al., 2013). Notably, AT_1_R may control this signaling pathway (Cingolani et al., 2013).

In response to sustained mechanical stress, the heart undergoes hypertrophic enlargement characterized by an increase in the size of individual cardiac myocytes. Although cardiac hypertrophy can initially be a compensatory response that temporarily augments and maintains cardiac output along with the Frank–Starling mechanism and the Anrep effect, prolonged hypertrophic stimuli can eventually lead to decompensation, heart failure, and arrhythmia (Levy et al., 1990; Ho et al., 1993). This pathological hypertrophy is induced by the activation of GPCRs by ligand or stretch stimulation, which in turn activates downstream signaling pathways, including mitogen-activated protein kinase (MAPK), protein kinase C (PKC), and calcineurin–nuclear factor of activated T cells (NFAT), leading to myocyte hypertrophy (Heineke and Molkentin, 2006). Candidates for control of this mechano-transduction of hypertrophic signaling include AT_1_R (Zou et al., 2004) and APJ (Scimia et al., 2012).

**FIGURE 2:**
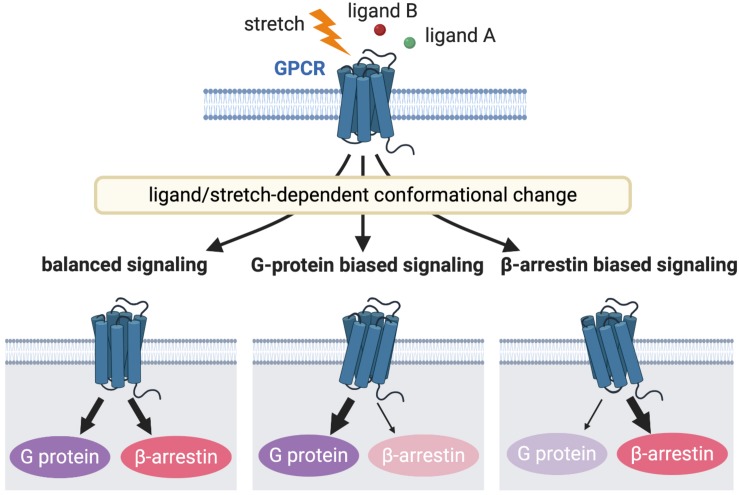
GPCR signaling induced by different receptor activation. Different stimulations stabilize the receptors into distinct active conformational states that couples to a particular G protein or β-arrestin or both to activate diverse arrays of downstream signaling, resulting in different functional outcomes.

The GPCR family is critical both at the bench and bedside, because the majority of current therapeutic drugs for heart failure target GPCRs (Lefkowitz, 2004). An expanding area of GPCR research is focused on the differential activation of G protein or β-arrestin signaling pathway in a “biased” manner to selectively promote cardiac beneficial pathways while preventing stimulation of cardiotoxic pathways. This biased agonism is achieved by ligands or mechanical stretch that can induce distinct active receptor conformations that in turn selectively activate only specific subsets of a given receptor (Figure 2) (Rakesh et al., 2010; Wisler et al., 2014). β-arrestin is a multifunctional scaffolding protein that desensitizes ligand-stimulated GPCRs but also can stimulate other signaling pathways distinct from G protein-dependent signaling (Reiter et al., 2012). Downstream of AT_1_R, chronic G protein-dependent signaling is associated with adverse outcomes, while β-arrestin-dependent signaling is considered beneficial for heart failure (Kim et al., 2012). Importantly, mechanical stress has been proposed to activate both G protein- and β-arrestin-dependent AT_1_R signaling pathways (Zou et al., 2004; Rakesh et al., 2010). In the APJ signaling system, in contrast, stretch stimulation selectively activates β-arrestin-dependent pathological pathway (Scimia et al., 2012), while apelin–APJ binding preferentially promotes G protein-dependent cardioprotective and prosurvival signaling. Because β-arrestins work as scaffolds that form complexes by binding to other proteins, it is conceivable that β-arrestins in AT_1_R and APJ show different functions due to their different binding partners. Thus, the role of these interacting pathways downstream of GPCRs in myocardial physiology appears to be receptor-dependent, and further investigation of how AT_1_R and APJ integrate ligand and mechanical stimuli to bias G protein or β-arrestin signaling, thus controlling cardioprotective versus cardiotoxic programs is important for the discovery of new therapeutics for heart failure.

This review aims to provide an up-to-date understanding of AT_1_R and APJ signaling pathways activated by mechanical stimuli on cardiac function and pathological hypertrophy, with special emphasis on biased stretch-mediated engagement of both AT_1_R and APJ and their potential roles in initiation or amelioration of heart diseases. The possibilities of targeting these pathways for the development of novel heart failure therapeutics will be discussed. Mechanistic insight will be provided through review of cell-based and animal models, and the areas of need for continued investigation will be highlighted.

## Angiotensin II Versus Stretch-Induced AT_1_R Signaling

Angiotensin II type 1 receptor plays pivotal roles in the regulation of cardiovascular function, and is one of the major targets for the therapeutic treatment of heart failure (Violin et al., 2013). Angiotensin II (Ang II), the endogenous ligand for AT_1_R, is well known for its action on vasoconstriction and aldosterone release, while its local action in the heart is also known to augment myocardial contraction (Petroff et al., 2000) and to activate hypertrophic signaling (Sadoshima and Izumo, 1993). It is widely accepted that myocytes respond to mechanical stretch to release Ang II in an autocrine fashion to activate AT_1_R and its intracellular signaling (Sadoshima et al., 1993). However, the receptor can also directly sense mechanical stress to activate its downstream signaling, even without Ang II binding (Zou et al., 2004; Yasuda et al., 2008; Rakesh et al., 2010). Komuro’s group showed for the first time that the mechanical activation of AT_1_R is agonist-independent in angiotensinogen-deficient mice (Zou et al., 2004). They also demonstrated that stretch stimulation of AT_1_R induces a conformational change of the receptor that is distinct from the ligand-activated receptor conformation (Yasuda et al., 2008). Despite some commonalities between stretch- and ligand-induced AT_1_R signaling pathways, recent studies have suggested that mechanical stress differentially activates β-arrestin-dependent AT_1_R signaling, which is distinct from ligand-activated pathways (Rakesh et al., 2010; Wang et al., 2018). This section summarizes the current understanding of how AT_1_R signaling regulates cardiac contractility and adaptive/maladaptive signaling when the heart is subjected to mechanical stress. In addition, the therapeutic possibility of targeting AT_1_R signaling for mechanical stress-relevant heart diseases is discussed.

### The Role of Ang II and AT_1_R in Ca^2+^-Independent Inotropic Response and the Frank–Starling Mechanism

Angiotensin II has a positive inotropic effect in cardiomyocytes both by myofilament Ca^2+^ sensitization and intracellular Ca^2+^ rise (Mattiazzi, 1997; Watanabe and Endoh, 1998). Experiments in isolated rabbit myocytes demonstrated that Ang II induced a positive inotropic effect without associated increases in either Ca^2+^ inward current or Ca^2+^ transients, but was accompanied by intracellular alkalosis that could potentially increase myofilament sensitivity to Ca^2+^ (Ikenouchi et al., 1994). It has also been shown that Ang II increases phosphorylation of myosin light chain 2 (MLC2) by the MLC–PKC pathway, thereby increasing myofilament Ca^2+^ responsiveness (Morano et al., 1988; Clement et al., 1992). This Ang II–AT_1_R activation of myofilaments could, therefore, form the basis for length-dependent activation (Frank–Starling mechanism). Indeed, Rockman’s group recently revealed that gene deletion or selective inhibition of AT_1_R in mouse hearts abrogates the Frank–Starling relationship (Abraham et al., 2016).

Using β-arrestin1/2 deficient mice, they also found that the loss of β-arrestin proteins abrogates Frank–Starling relationship without activating PKC. It has been demonstrated that β-arrestin-biased AT_1_R activation enhances myocyte contractility without increasing intracellular Ca^2+^ concentration (Rajagopal et al., 2006), and myofilament Ca^2+^ sensitivity associated with reduced TnI and MyBPC phosphorylation and enhanced tropomyosin phosphorylation (Monasky et al., 2013; Ryba et al., 2017). However, protein phosphorylation in the β-arrestin1/2 deficient mice does not differ compared to wildtype controls. This may be due to distinct β-arrestin signaling pathways downstream of stretch–AT_1_R compared to ligand–AT_1_R stimulation (Wang et al., 2018). Further detailed investigation of posttranslational modifications of myofilament proteins by proteomic analyses will identify novel proteins critical to the AT_1_R-dependent modulation of the Frank–Starling relationship.

### The Role of AT_1_R in Ca^2+^-Dependent Inotropic Response and Slow Force Response

**FIGURE 3:**
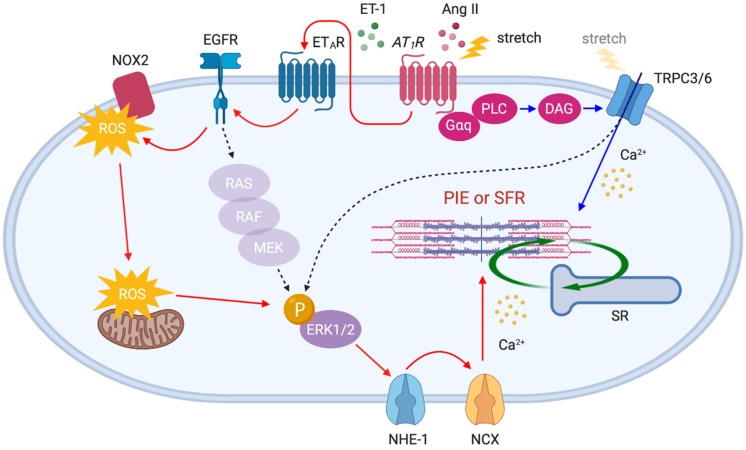
AT_1_R downstream signaling pathways activated by acute stretch or Ang II. Membrane stretch directly or indirectly (by the autocrine release of Ang II) stimulates AT_1_R which in turn activates distinct downstream signaling pathways: (1) ET_*A*_R–EGFR–ERK1/2–NHE-1–NCX axis (red arrows) and (2) PLC–DAG–TRPC3/6 axis (blue arrows). The gradual influx of Ca^2+^ through NCX and TRPC3/6 channels enhances myocardial contractility, thereby initiating SFR. Ang II-triggered positive inotropic effect (PIE) shares the same pathways.

In addition to the positive inotropic effect caused by myofilament Ca^2+^ sensitization, Ang II enhances contractility by a Ca^2+^ dependent mechanism. It has been proposed that Ang II–AT_1_R binding triggers endothelin-1 (ET-1) production/release, which in turn activates endothelin type A receptor (ET_*A*_R) to induce transactivation of epidermal growth factor receptor (EGFR) through ROS-induced ROS release (Cingolani et al., 2006; Yeves et al., 2015; Zhang et al., 2015). This results in NHE-1 activation to induce Na^+^ influx that in turn triggers Ca^2+^ entry via reverse mode NCX, thereby enhancing contractility (Petroff et al., 2000; Pérez et al., 2003) (Figure 3, red arrows). It has been suggested that these mechanisms could be the basis for the SFR (the *in vitro* equivalent of the Anrep effect). Cingolani’s group has described a similar complex signaling pathway upon stretch-induced Ang II production, involving autocrine/paracrine activation of AT_1_R and ET_*A*_R (Cingolani et al., 2011), ROS-induced ROS release (Caldiz et al., 2007), the transactivation of EGFR (Brea et al., 2016), ERK1/2 activation (Pérez et al., 2011), and NHE-1 and NCX activation (Pérez et al., 2001), thus increasing myocyte contraction (Figure 3, red arrows).

Recently, we demonstrated that transient receptor potential canonical (TRPC) 6 channel is an additional important upstream determinant of SFR. Our study demonstrated that gene deletion and selective blockade of TRPC6 channel abrogates SFR and the Anrep effect in physiologically loaded myocytes, muscle strips, and intact hearts (Seo et al., 2014a). More recently, it was found that TRPC3 is an equivalent contributor to this process (Yamaguchi et al., 2018) as supported by evidence that TRPC3 and 6 can form functional heterotetramers (Hofmann et al., 2002). The mechanosensitivity of TRPC6 had been demonstrated in smooth muscle cells (Spassova et al., 2006) and adult cardiomyocytes (Dyachenko et al., 2009) exposed to membrane stretch or shear stress. Some, however, have questioned the mechanosensing capacity of TRPC6 and attributed it to its upstream stretch-activated GPCRs and/or artifacts from TRPC6 overexpression in heterologous systems (Gottlieb et al., 2008). Importantly, TRPC3/6 is a downstream component of AT_1_R, and Ang II stimulation is known to activate these channels via G_α__*q*_ protein, PLC and diacylglycerol (DAG) signaling pathway (Onohara et al., 2006). Indeed, stretch-induced slow increase in Ca^2+^ was suppressed by either TRPC3/6 or AT_1_R inhibition (Yamaguchi et al., 2018). Ca^2+^ influx through TRPC3/6 may directly stimulate SFR, while it is also conceivable that TRPC3/6 activates ERK1/2 (Yao et al., 2009; Chiluiza et al., 2013) upstream of NHE-1, thus inducing an inward Na^+^ current that in turn triggers Ca^2+^ entry via NCX (Poburko et al., 2007, 2008; Louhivuori et al., 2010) (Figure 3, blue arrows). While many of these studies have suggested the involvement of AT_1_R as an upstream component of SFR signaling pathways, there are several opposing reports describing that Ang II and AT_1_R are not involved in the process because SFR was not suppressed by Ang II receptor blockers (ARBs) (Calaghan and White, 2001; von Lewinski et al., 2003; Shen et al., 2013). Despite the inverse agonistic effect of ARBs (Sato et al., 2016), mechanical stretch may differentially activate AT_1_R even in the presence of ARBs which should normally suppress the ligand-stimulated signaling pathway. More direct evidence that links AT_1_R to SFR, using AT_1_R-deficient models, will be needed to clarify this mechanism.

### Pathological AT_1_R–TRPC3/6 and Cardioprotective AT_1_R–β-Arrestin Signaling in Pressure-Overload Cardiac Hypertrophy

It is well established that Ang II can induce cardiomyocyte hypertrophy through the activation of multiple intracellular signaling pathways such as the mitogen-activated protein kinase (MAPK) signaling cascade (Thorburn et al., 1994; Zechner et al., 1997; Wang et al., 1998a), c-Jun *N*-terminal kinase (Sadoshima and Izumo, 1993; Ramirez et al., 1997; Wang et al., 1998b), Akt–mammalian target of rapamycin (mTOR) (Gorin et al., 2001; Diniz et al., 2009) and calcium–calmodulin-activated phosphatase calcineurin (Molkentin et al., 1998; Wilkins and Molkentin, 2004). In particular, calcineurin is a pivotal regulator of pathological cardiac hypertrophy preferentially activated by mechanical stress on GPCRs (Molkentin, 2004; Heineke and Molkentin, 2006). Once activated by increases in Ca^2+^, calcineurin mediates the hypertrophic response through its downstream transcriptional effector NFAT (Crabtree and Olson, 2002). It has been accepted that a major source of Ca^2+^ for activation of calcineurin is Ca^2+^ influx through TPRC3 and 6 channels. Indeed, Ang II–AT_1_R activation promotes calcineurin–NFAT signaling that requires the DAG-induced Ca^2+^ signaling pathway through TRPC3 and 6 (Onohara et al., 2006). Importantly, this signaling pathway can be activated by mechanical stress. TRPC3 overexpressed transgenic mice exhibit an increase in calcineurin–NFAT activation *in vivo*, and increased hypertrophy after Ang II/Phenylephrine and pressure-overload stimulation (Nakayama et al., 2006). In addition, TRPC6 transgenic mice also resulted in enhanced sensitivity to mechanical stress, with an increase in calcineurin–NFAT signaling, and severe cardiac hypertrophy and failure (Kuwahara et al., 2006). Although TRPC3/6 channels are linked to other G_α__*q*_PCRs such as ET_*A*_R and α-adrenergic receptors, AT_1_R may be the putative central actor in stress-induced hypertrophy, considering the mechanosensing capacity of the receptor.

AT_1_R activation by either Ang II or mechanical stress not only induces pathological signaling but also promotes physiological hypertrophy and prosurvival signaling. In embryonic, neonatal, and adult cardiomyocytes, Ang II–AT_1_R activation promotes transactivation of EGFR, which in turn activates MAPK and Akt–mTOR pathways (Thomas et al., 2002; Diniz et al., 2009). Rockman’s group recently demonstrated that mechanical stress in cells and the hearts activates AT_1_R-induced prosurvival signaling in a β-arrestin-dependent manner that does not require Ang II release (Rakesh et al., 2010). The formation of an AT_1_R–β-arrestin complex by mechanical stress induces EGFR transactivation and subsequent ERK and Akt signaling pathways, which suppresses cardiomyocyte injury (Rakesh et al., 2010). They later found that membrane stretch uniquely promotes the coupling of the inhibitory G protein (G_α__*i*_) that is required for the recruitment of β-arrestin2 and activation of downstream signaling pathways, such as EGFR transactivation and ERK phosphorylation (Wang et al., 2018). G_α__*i*_ proteins primarily inhibit the cAMP-dependent pathway by inhibiting adenylyl cyclase (AC) activity. Although previous studies have shown that Ang II may promote AT_1_R–G_α__*i*_ coupling to inhibit AC and to regulate Ca^2+^ channels in certain tissues or cell types (Hescheler et al., 1988; Maturana et al., 1999), it is not yet clear if the G_α__*i*_–β-arrestin complex has the same function.

**FIGURE 4:**
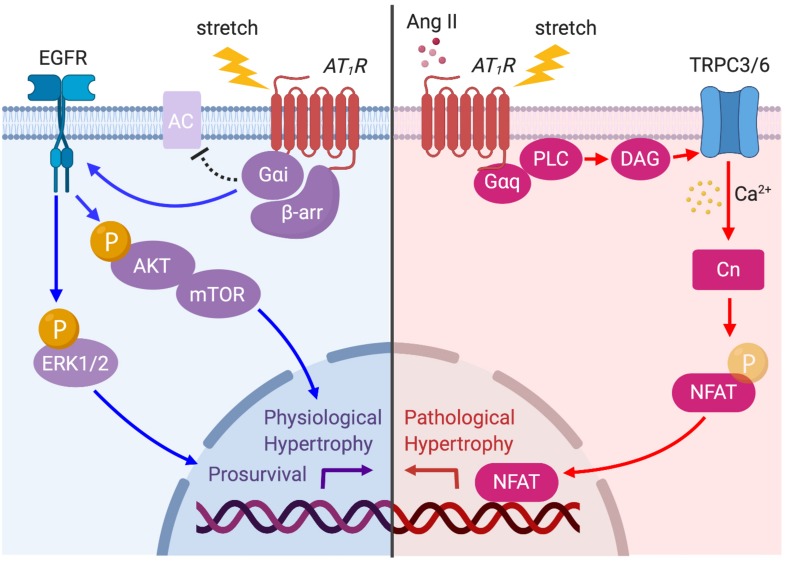
AT_1_R signaling pathways activated by prolonged mechanical load or Ang II. The AT_1_R is principally coupled to G_α__*q*_ protein but is also coupled to G_α__*i*_ protein through β-arrestin (β-arr) recruitment when the myocyte is subjected to mechanical load. Ang II- or stretch-induced G_α__*q*_ signaling activates TRPC3/6 to initiate Ca^2+^ influx, which in turn activates calcineurin (CN)–NFAT pathway to promote pathological cardiac hypertrophy (red arrows). AT_1_R–G_α__*i*_ coupling induces EGFR transactivation and Akt/ERK phosphorylation, which promotes physiological hypertrophy and prosurvival signaling (blue arrows).

Taken together, chronic mechanical stress can induce both G_α__*q*_–TRPC3/6 dependent and G_α__*i*_–β-arrestin dependent signaling pathways to differentially promote pathological and prosurvival signaling (Figure 4). Although selective G_α__*q*_–TRPC3/6 signaling initially works as an adaptive response to mechanical stress by enhancing myocardial contractility through SFR, prolonged signaling eventually worsens cardiac function. On the other hand, β-arrestin-dependent AT_1_R signaling is proposed to enhance cardiac contractility by a Ca^2+^-independent mechanism, and chronically activates prosurvival signaling, making it a pathway of high clinical potential to ameliorate acute and chronic heart failure.

### AT_1_R Targeted Therapeutics for Mechanical Stress-Associated Heart Diseases (Table 1)

Pathological hypertrophy induced by the overstimulation of AT_1_R by Ang II or mechanical stress can eventually lead to heart failure and sudden death associated with arrhythmia. One of the current therapeutics for these conditions is AT_1_R blocking drugs, known as ARBs. Several ARBs are known to have inverse agonistic action which can inactivate the GPCR state, and thereby suppress the constitutive activity of receptors. Such drugs can suppress mechanical stretch-induced signals through AT_1_R and may exhibit enhanced therapeutic effects for these disease states (Zou et al., 2004; Wei et al., 2011).

**TABLE 1:** Emerging drugs for heart failure therapeutics targeting AT_1_R or APJ.

**Name of drug**	**Drug class**	**Model studied/clinical trials**
TRV120027	β-arrestin-biased AT_1_R agonist	ADHF (Phase 2B BLAST-AHF, no benefit over placebo)
TRV120023	β-arrestin-biased AT_1_R agonist	DCM (mice, improved contractility)
		ACI (mice/rats, improved contractility, reduced cell death)
TRV120067	β-arrestin-biased AT_1_R agonist	DCM (mice, improved contractility and structure)
Sildenafil	PDE5 inhibitor	HFpEF (Phase 3 RELAX, no benefit over placebo)
		HFrEF (Phase 3 SIL-HF, recruiting)
BI 749327	TRPC6 inhibitor	CHF (mice, improved LV function and reduced fibrosis)
ML233	small molecule APJ agonist	No reports on HF
AMG 986	small molecule APJ agonist	CHF (Phase 1, terminated)
MM07	G-protein-biased peptide APJ agonist	No reports on HF
CLR325	peptide APJ agonist	CHF (Phase 2, completed)

*ADHF, acute decompensated heart failure; DCM, dilated cardiomyopathy; ACI, acute cardiac injury; CHF, chronic heart failure.*

While endogenous Ang II activates both G protein and β-arrestin signaling pathways, several AT_1_R ligands, such as [Sar1, Ile4, Ile8]-Ang II (SII), TRV120023, TRV120027, and TRV120067 have been shown to selectively activate β-arrestin-mediated pathways and therefore termed as β-arrestin-biased agonists (Rajagopal et al., 2010; Strachan et al., 2014). These β-arrestin-biased AT_1_R agonists have been demonstrated to have beneficial effects on the disease condition caused by mechanical stress. For example, TRV120027 causes cardiac unloading action while preserving renal function in a canine model of acute heart failure (Boerrigter et al., 2011). However, a Phase 2B trial of TRV120027 in acute heart failure (BLAST-AHF) resulted in no benefit over placebo (De Vecchis et al., 2017). On the other hand, TRV120023 diminishes myocyte apoptosis caused by mechanical stress by selectively activating ERK1/2 cardioprotective signaling pathways in isolated mouse hearts (Kim et al., 2012). Long-term treatment with TRV120067 in the mouse model of dilated cardiomyopathy not only prevented maladaptive signaling but also improved cardiac function by altering the myofilament response to Ca^2+^ via β-arrestin signaling pathways (Ryba et al., 2017). Notably, TRV120023 and TRV120067 have shown better efficacy in cardiac function and cardioprotection compared to ARBs, suggesting their possibility to become novel therapeutic drugs for heart failure.

Targeting AT_1_R downstream signals, such as protein kinase G (PKG) and TRPC3/6, could be another therapeutic strategy. Our study previously revealed that adverse myocardial responses induced by mechanical stimulation to these channels are suppressed by post-transcriptional modification of TRPC6 channel by activation of the cGMP–PKG pathway (Seo et al., 2014a). Indeed, there is growing evidence that stimulation of the cGMP–PKG pathway within cardiac myocytes dampens cardiac stress responses, and its activation can attenuate pathological hypertrophy, protect against ischemic injury and enhance cell survival (Zhang and Kass, 2011). One means to activate the cGMP–PKG pathway is to inhibit the degradation of cGMP by phosphodiesterase-5 (PDE5). PDE5 inhibitors [e.g., sildenafil (Viagra)] have proven their efficacy in treating pressure-overload cardiac hypertrophy and failure in animal models (Takimoto et al., 2005; Nagayama et al., 2009). Although the RELAX trial, a large clinical trial of PDE5 inhibition in heart failure with preserved ejection fraction (HFpEF), failed to show robust beneficial effects (Redfield et al., 2013), single-center trials in patients with heart failure with reduced ejection fraction (HFrEF) reported improved exercise capacity and quality of life (Lewis et al., 2007). Indeed, a meta-analysis of randomized controlled trials in heart failure shows statistically significant improvement of clinical outcomes in patients with HFrEF rather than HFpEF (De Vecchis et al., 2017). Our study demonstrated that sildenafil attenuates pathological hypertrophy by promoting TRPC6 phosphorylation by PKG in the mouse model of muscular dystrophy in which the heart is susceptible to mechanical load (Seo et al., 2014a). Direct antagonism of TRPC3/6 channels is also proven effective for preventing pathological hypertrophy in experiment levels (Seo et al., 2014b). Recently, the orally bioavailable selective TRPC6 inhibitor (BI 749327) was tested in mice, providing *in vivo* evidence of therapeutic efficacy for cardiac and renal stress-induced disease with fibrosis (Lin et al., 2019). Thus, direct inhibition of TRPC6 could be an alternative strategy to effectively suppress pathological cardiac hypertrophy and failure induced by adverse mechanical stress.

## Apelin Versus Stretch-Induced APJ Signaling

Apelin receptor is a GPCR that binds the endogenous peptide apelin (Tatemoto et al., 1998; Lee et al., 2000). This receptor is widely expressed in the cardiovascular system and is emerging as an important mediator of both cardiac and vascular function (Chandrasekaran et al., 2008). In the heart, apelin has been shown to increase myocardial contraction (Szokodi et al., 2002), reduce cardiac load (Ashley et al., 2005) and promote cardioprotective effects (Zhang et al., 2009). Recently, a peptide named ELABELA (Ryba et al., 2017) or Toddler (Pauli et al., 2014) was found to bind APJ, exhibiting a cardiac protective role comparable to apelin (Sato et al., 2017). In addition to this new ligand, it recently turned out that mechanical stretch can also directly activate this receptor. It has been proposed that APJ acts as a bifunctional receptor for both mechanical stress and apelin to activate separate signaling pathways directed to inotropic, cardiotoxic, and cardioprotective effects (Scimia et al., 2012). Intriguingly, unlike AT_1_R, stretch-induced activation of APJ triggers pathological hypertrophy through β-arrestin, while β-arrestin-dependent signaling in AT_1_R activates prosurvival signaling. This section summarizes the up-to-date findings of APJ signaling pathways stimulated by apelin or mechanical stress, and introduces how each stimulation can bring different cardiac outcomes. Therapeutic possibilities of biased agonists for heart failure targeting G protein versus β-arrestin-dependent signaling pathways are discussed.

### The Role of Apelin–APJ in Ca^2+^-Independent Positive Inotropic Effect and Frank–Starling Relationship

A potent inotropic effect of apelin has been demonstrated in cardiomyocytes (Farkasfalvi et al., 2007; Wang et al., 2008; Peyronnet et al., 2017), muscle strips (Dai et al., 2006), isolated hearts (Szokodi et al., 2002; Perjés et al., 2014) and *in vivo* heart disease models (Berry et al., 2004; Charo et al., 2009). It has been proposed that the increase in myocardial contractility is attributed to both Ca^2+^-dependent (Dai et al., 2006; Wang et al., 2008) and Ca^2+^-independent mechanisms (Farkasfalvi et al., 2007; Charo et al., 2009; Parikh et al., 2018). The latter is considered to rely on apelin’s action on myofilament sensitivity to Ca^2+^. It was first demonstrated by Farkasfalvi et al. (2007) in studies with normal and failing cardiomyocytes that displayed increased sarcomere shortening in the absence of increased Ca^2+^ transient amplitude after apelin administration. They suggested one of the mechanisms involves increased myofilament sensitivity to Ca^2+^ as apelin activates NHE-1 with consequent intracellular alkalinization. Subsequently, a study using isolated single left ventricular myocytes from apelin deficient and APJ deficient mice revealed that loss of apelin or APJ causes impaired contraction with no difference in intracellular Ca^2+^ kinetics, suggesting apelin and APJ affect either myofilament Ca^2+^ sensitivity or cross-bridge cycling kinetics (Charo et al., 2009). This may be attributable to the activation of MLC kinase through parallel and independent activation of PKCε and ERK1/2 signaling stimulated by apelin (Perjés et al., 2014).

**FIGURE 5:**
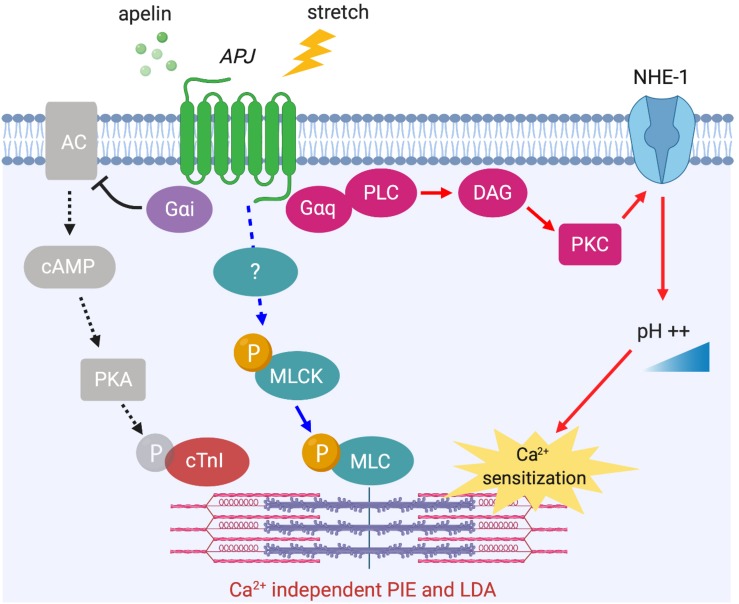
APJ downstream signaling pathways for Ca^2+^ independent positive inotropic effect (PIE) and length-dependent activation (LDA). Apelin-induced activation of APJ enhances myofilament sensitivity to Ca^2+^, which leads to the positive regulation of LDA. The mechanisms include intracellular alkalinization by G_α__*q*_-dependent NHE-1 activation (red arrows), G_α__*i*_-dependent dephosphorylation of cTnI (black dashed arrows), and the phosphorylation of MLC (blue arrows, mechanism unknown).

The phosphorylation level of myofilament proteins can affect length-dependent activation, which positively regulates the Frank–Starling relationship. Recently, the direct effect of apelin on length-dependent activation was examined in mechanically preloaded cardiomyocytes; apelin increased compliance of the myocytes as indicated by the negative regulation of end-diastolic force-length relationship, which in turn enhanced contractility as indicated by increased Frank–Starling gain (dimensionless index for contractility) (Peyronnet et al., 2017). Increased cardiomyocyte compliance is presumably related to titin phosphorylation. However, the observed positive regulation of the Frank–Starling relationship by apelin must be dependent on other contractile proteins such as cTnI and MLC or alkalosis, because decreased titin-based stiffness is associated with reduced length-dependent activation of myocardium (Methawasin et al., 2014; Ait-Mou et al., 2016; Beqqali et al., 2016). Recently, we demonstrated that APJ-deficient cardiomyocytes showed negative regulation of the Frank–Starling relationship with no increase in Ca^2+^ transients in response to stretch (Parikh et al., 2018). Our study also provided mechanistic insights for apelin’s positive inotropic and Frank–Starling effects. We demonstrated reduced protein kinase A (PKA) phosphorylation of cTnI at Ser^22^/Ser^23^ in response to apelin. This is known to increase myofilament Ca^2+^ sensitivity, and is consistent with apelin-dependent AC–cAMP–PKA inhibition through G_α__*i*_ activation (Szokodi et al., 2002; Scimia et al., 2012) (Figure 5).

### The Role of Apelin–APJ in Ca^2+^-Dependent Positive Inotropy and the Anrep Effect

**FIGURE 6:**
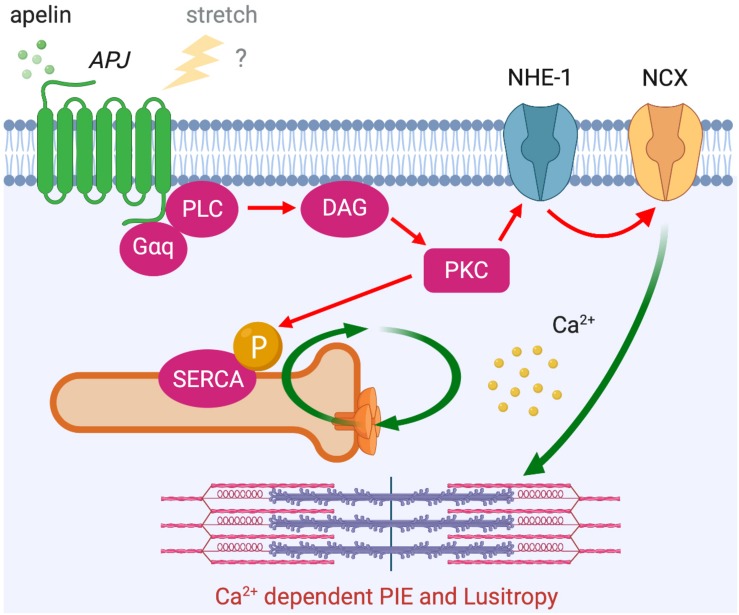
APJ downstream signaling pathway for Ca^2+^ dependent positive inotropic effect (PIE). APJ activation by apelin also induces PIE that accompanies increased Ca^2+^ transient through G_α__*q*_-dependent NCX activation (red arrows). Positive lusitropic effect (increased velocity of myocardial relaxation) is also induced via PKC-directed phosphorylation of SERCA.

In addition to this Ca^2+^-independent mechanism, apelin is thought to exert its inotropic action by increasing the availability of intracellular Ca^2+^. Apelin’s inotropic effect in isolated hearts is dependent on PLC, PKC, NHE-1, and NCX activation (Szokodi et al., 2002). Notably, this inotropic response develops slowly to reach a plateau within 10–30 min and is then sustained, which is different from classical β-adrenergic receptor activation that develops rapidly over a matter of seconds. Although Ca^2+^ dynamics were not examined in this study, a later study proposed that apelin-induced increased contractility is the result of increased Ca^2+^ transients rather than changes in myofilament Ca^2+^ responsiveness (Dai et al., 2006). Most recently, these observations were consolidated in a report showing that apelin has positive inotropic and lusitropic actions on isolated myocytes with enhanced calcium-induced calcium release. This enhanced Ca^2+^ release is achieved through increased Ca^2+^ influx through NCX and increased rate of Ca^2+^ uptake to Ca^2+^ storage by sarcoplasmic reticulum Ca^2+^-ATPase (SERCA), as controlled by PKC-directed phosphorylation (Wang et al., 2008). It is intriguing that the time course and the signaling pathways underlying the effect of apelin show some similarities to the mechanism of SFR (Figure 6 compared to Figure 3), in which NHE-1 and NCX are the primary downstream actors. Although the role of APJ in SFR has yet to be fully explored, it is conceivable that this signaling pathway modulates this physiological phenomenon both through apelin–APJ binding and APJ’s mechanosensing ability.

### Integration of Apelin and Mechanical Stimuli in APJ to Differentially Activate Cardiac Prosurvival and Hypertrophic Signaling

We previously demonstrated a beneficial effect of chronic apelin supplementation on cardiac performance with reduced left ventricular preload and afterload, and increased contractile function without evidence of hypertrophy (Ashley et al., 2005). Chronic stimulation of APJ by apelin not only increases cardiac performance but also attenuates the development of pressure-overload heart failure through the inhibition of TGF-β-driven profibrotic activity (Pchejetski et al., 2012) and NOX2-derived ROS production (Koguchi et al., 2012). In apelin-deficient mice, hypertrophic response to pressure overload was unchanged, but the progressive impairment of systolic function was observed (Kuba et al., 2007). Myocardial infarcted hearts in apelin deficient mice exhibit exacerbated postinfarction remodeling and impaired functional recovery with a significant reduction of prosurvival phospho-Akt and ERK1/2 signals in the infarct and peri-infarct regions (Wang et al., 2003). These studies clearly demonstrate the simultaneous inotropic and antihypertrophic effects of apelin in ischemia and pressure overload.

**FIGURE 7:**
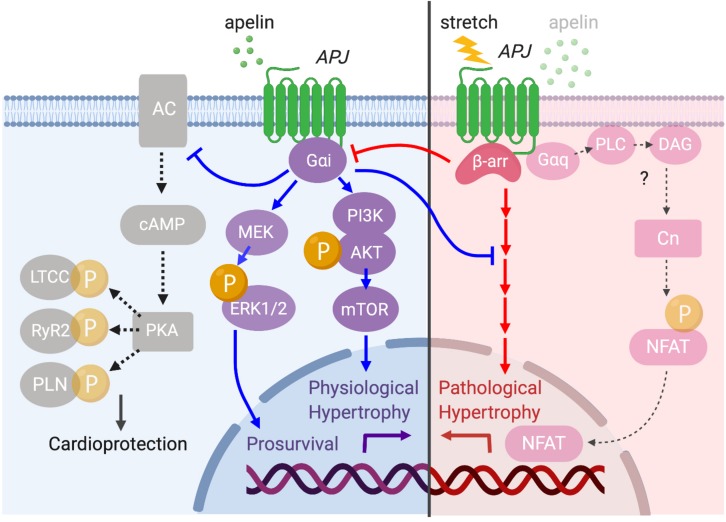
Diverse APJ signaling pathways activated by prolonged mechanical load or apelin. APJ translates apelin and stretch stimuli into distinct downstream signaling pathways. Apelin stimulation induces G_α__*i*_-dependent signaling to promote physiological hypertrophy and prosurvival signaling through the activation of Akt–mTOR and ERK1/2 (blue arrows). G_α__*i*_ also inhibits deleterious cAMP–PKA pathway to serve a cardioprotective role (black dashed arrows). On the other hand, membrane stretch activates a β-arrestin-dependent program that results in pathological hypertrophy although the detailed downstream mechanisms are unresolved (red arrows). Importantly, there is a functional interplay between apelin- and stretch-dependent APJ signaling, in which apelin–APJ signaling blunts stretch-induced pathological signaling while stretch–APJ signaling interferes with apelin-induced G_α__*i*_ activation. Although the apelin–APJ system involves G_α__*q*_-dependent signaling pathway (Figure 6), the relevance of this pathway to pathological hypertrophy has not been examined.

Conversely, cardiomyocyte-specific overexpression of APJ causes cardiac hypertrophy and contractile dysfunction in mice (Murata et al., 2016). This indicates that APJ has a capacity to activate multiple downstream signaling pathways such as G_α__*i*_- and G_α__*q*_-dependent pathways (Chapman et al., 2014), some of which may be independent of apelin’s protective effects. It has also been shown that APJ can integrate chemical (apelin) and mechanical (stretch) stimuli and translates these into opposite cardiac outcomes by differentially activating downstream pathways (Scimia et al., 2012). Specifically, apelin activates APJ through G_α__*i*_ protein to exert its cardioprotective effect, while stretch stimulates APJ to recruit β-arrestins, which promote pathological hypertrophy (Figure 7). At the cellular level, the mechanosensing capacity of APJ has been confirmed in H9c2 cardiomyocytes in which the increase in diameter, volume and protein content of cardiomyocytes under static pressure was ameliorated by APJ shRNA (Xie et al., 2014). Recently, our study provided supporting evidence that myocyte-specific deletion of APJ is protective against pressure-overload heart failure, showing the abrogation of mechanosensing capacity, reduced Ca^2+^ transient, and remarkable suppression of cellular hypertrophy and fibrosis (Parikh et al., 2018). These studies suggest that APJ integrates apelin and stretch stimuli, biasing the level of G protein versus β-arrestin signaling to attenuate or stimulate hypertrophy. While the downstream mechanisms of stretch-induced β-arrestin signaling remain undefined, the function of β-arrestins has also been observed to block the interaction of APJ with G_α__*i*_ proteins (i.e., desensitization), which may contribute to the pro-hypertrophic program (Scimia et al., 2012). These studies have implications for the consideration of APJ as a drug target, because the greatest benefit may be obtained not simply by apelin stimulation, but rather by selectively activating the G_α__*i*_-dependent signaling pathway or by inhibiting the ability of APJ to respond to mechanical stretch. For this purpose, further efforts are critical to clarify the intricacies of downstream integration of ligand and mechanosensitive signaling by APJ.

### APJ Targeted Therapeutics for Mechanical Stress-Associated Heart Diseases (Table 1)

Apelin’s positive inotropy and anti-hypertrophic effects support its therapeutic potential in preventing and treating cardiovascular disease. The majority of current heart failure therapies are targeted at the inhibition of deleterious neurohormonal axes that are upregulated in the later stages of the disease. In this aspect, the apelin–APJ system is attractive because it appears to be downregulated in heart failure (Japp and Newby, 2008), and stimulation of this pathway may have additive or even synergistic efficacy to current therapy by targeting complementary but separate pathways. Indeed, intravenous administration of [Pyr^1^] apelin-13, an active fragment of apelin, in heart failure patients showed efficacy with peripheral and coronary vasodilatation and increases in cardiac output (Japp et al., 2010).

Nonetheless, the therapeutic application of apelin is limited because of its extremely short biological half-life and parenteral administration. This is attributed to the degradation by endogenous proteases circulating in the blood (Japp et al., 2008; Zhen et al., 2013). Furthermore, apelin is hydrolyzed and partially deactivated by angiotensin I converting enzyme 2 (ACE2) (Vickers et al., 2002; Wang et al., 2016). Thus, many efforts have been directed to the development of apelin analogs or novel agonists of APJ (Yamaleyeva et al., 2016). In particular, combinatorial screening of the NIH small molecule library identified a full non-peptide APJ agonist, ML233, at activating both G_α__*i*_- and β-arrestin-dependent pathways (Khan et al., 2011). In addition, using molecular dynamics simulations, Brame et al. (2015) designed cyclic analogs and identified a biased agonist, MM07, which activates APJ by preferentially stimulating G_α*i*_-dependent pathways but not β-arrestin. Because stimulation of β-arrestin pathway could be deleterious, G protein-biased APJ ligand MM07 represents a potential new therapeutic for heart failure.

## Stretch-Induced Conformational Change in AT_1_R and APJ

G protein-coupled receptors adopt distinct conformations to selectively activate different arrays of downstream signaling (Rosenbaum et al., 2009; Vaidehi and Kenakin, 2010). Detailed analysis of the conformational changes is often performed by crystal structure analysis, but this only allows a static view of a given receptor conformation. Bioluminescence resonance energy transfer (BRET) and fluorescence resonance energy transfer (FRET) techniques allow insight into the kinetics and amplitudes of agonist- and stretch-induced receptor conformations. Using these techniques, stretch-induced conformational changes of AT_1_R have been studied. BRET revealed that membrane stretch induces an active AT_1_R conformation allowing for G protein activation and subsequent β-arrestin recruitment (y Schnitzler et al., 2008). More recently, a study using BRET and FRET demonstrated that G protein activation is not necessary for β-arrestin recruitment and that mechanical stretch induces a particular β-arrestin conformation that is distinct from the agonist-stimulated conformation (Rakesh et al., 2010). Another approach was also taken to examine the conformational change of AT_1_R in response to stretch. Using the substituted cysteine accessibility method (SCAM) and molecular modeling approach, Yasuda et al. (2008) showed that stretch stimulation of AT_1_R induces a dislocation and a counterclockwise rotation of transmembrane domain 7 toward the ligand-binding pocket, a conformation that is distinct from the ligand-activated receptor conformation. These fascinating data suggest that conformational change is responsible for biased signaling in AT_1_R. Such studies of APJ and examination of the differential effect of novel AT_1_R ligands on its conformation could eventually lead to structure-based drug design focused on selective inhibition of adverse remodeling via stretch-dependent pathways.

## Interaction Between Ang-II–AT_1_R and Apelin–APJ

Neurohormonal interaction between AT_1_R and APJ systems has been well-studied. Infusion of Ang II in rats causes an acute reduction of apelin and APJ levels in the heart, which is abolished by treatment with an ARB (Iwanaga et al., 2006). The left ventricular dysfunction observed in apelin deficient mice is restored to normal levels either by the treatment with an ARB or by AT_1_R gene deletion (Sato et al., 2013). Furthermore, apelin overexpression abolishes Ang II-induced cardiac hypertrophy in cultured myocytes (Ye et al., 2015). While a growing literature suggests the antagonistic interplay between AT_1_R and APJ systems through neurohormonal interactions, direct physical interaction between AT_1_R and APJ has also been reported. Co-immunoprecipitation and FRET analysis revealed heterodimerization of AT_1_R and APJ, in which the interaction appears to induce the inhibition of the AT_1_R signaling pathway (Chun et al., 2008). More recently, it was revealed that AT_1_R–APJ heterodimerization is induced by apelin, but is not affected by Ang II (Siddiquee et al., 2013). To date, the downstream effects of ligand and stretch stimulation on heterodimeric AT_1_R–APJ have not been examined. It is conceivable that AT_1_R–APJ heterodimers activate distinct signaling pathways compared to monomeric receptors, or that the multiple reported downstream pathways of AT_1_R and APJ may be partially dependent on the activation of the heterodimeric receptor. Understanding of this may lead to the identification of novel ligands with optimal effects on heart failure pathobiology.

## Conclusion

Angiotensin II type 1 receptor and APJ are mechanosensitive GPCRs in the heart, playing vital roles in cardiac physiological adaptation to changes in mechanical load. However, when a mechanical load is excessive and sustained, it can induce maladaptive hypertrophic signaling. Enormous effort has been invested in understanding the physiological and pathophysiological roles of AT_1_R and APJ signaling to identify novel therapeutic strategies. Selective activation or inhibition of mechanically stimulated signaling components by biased agonists may yield more precise molecular enhancers of desired inotropic or cardioprotective effects while avoiding detrimental signaling. In addition, by gaining an improved understanding of the signaling mechanisms of these receptors in the heart, it is likely that complementary downstream targets will be identified to modulate cardiac function and ameliorate disease.

## Author Contributions

KS took the lead in writing the manuscript with input from VP and EA.

## Conflict of Interest

The authors declare that the research was conducted in the absence of any commercial or financial relationships that could be construed as a potential conflict of interest.
